# Lumbar disc herniation modelling: a review of ex-vivo mechanical models and a comparison with clinical data

**DOI:** 10.1007/s00586-025-09054-x

**Published:** 2025-06-25

**Authors:** Thomas David Slater, Hans-Joachim Wilke, Gnanaprakash Gurusamy, Shanmuganathan Rajasekaran, Nicolas Newell

**Affiliations:** 1 Department of Bioengineering, Imperial College London, London, United Kingdom; 2 Institute of Orthopaedic Research and Biomechanics, University of Ulm, Ulm, Germany; 3 Department of Orthopaedics and Spine Surgery, Ganga Hospital, Coimbatore, Tamil Nadu India

**Keywords:** Intervertebral disc, Herniation, Spine, Ex-vivo, Lumbar

## Abstract

**Purpose:**

Ex-vivo herniation models are essential for studying lumbar disc herniation mechanisms, but their morphological accuracy remains unclear due to limited validation against patient-derived clinical data. This review collates clinical lumbar disc herniation characteristics and evaluates whether existing models replicate real-world pathology. By identifying the most morphologically relevant models, this study provides a stronger foundation for improving mechanistic herniation models.

**Methods:**

A systematic review following PRISMA guidelines identified clinical studies detailing herniation characteristics and experimental models of ex-vivo lumbar disc failure. Models were categorised by loading conditions (complex ultimate compression; cyclic; and intradiscal pressurisation), then compared to clinical data to assess their validity.

**Results:**

In patients, extrusions (50%) and protrusions (34%) are the most common lumbar disc herniation types, with paracentral herniations (61%) predominantly occurring at L4-L5 (49%) and L5-S1 (42%). Structural failure patterns varied, with annulus fibrosus failure reported in 35–81% of cases and endplate junction failure in 19–68%.

Among 25 analysed models, all loading types induced herniations, but often with different damage patterns. Complex ultimate compression caused abrupt failures and fractures, while cyclic led to progressive annular damage. Intradiscal pressurisation highlighted nucleus pulposus migration pathways. Within a single herniation model, the damage mechanisms seen were similar between discs.

**Conclusions:**

Clinical herniation patterns show significant variability, while ex-vivo models yield more repeatable outcomes. Cyclic, complex ultimate compression, and intradiscal pressurisation models provide valuable mechanistic insights but differ in physiological relevance. Researchers must consider the physiological relevance of the applied load and the differences between animal and human discs when selecting a model. Future research should focus on understanding herniation progression and identifying initiating factors to improve prevention strategies.

**Supplementary Information:**

The online version contains supplementary material available at 10.1007/s00586-025-09054-x.

## Background

Intervertebral discs (IVD) consist of a soft, gel-like nucleus pulposus (NP) at the centre, surrounded by strong annulus fibrosus tissue (AF). The annulus fibrosus consists of several layers of concentric lamellae (15–25), each made of collagen fibres that are oriented obliquely to the transverse plane (approximately 30°), providing structural integrity and resisting tension and shear forces [1]. The NP and AF are bordered cranially and caudally by cartilaginous and bony endplates (EP), which anchor the disc to the adjacent vertebral bodies [2]. They form an interface between vertebral bodies and discs that helps transfer forces, facilitate nutrition exchange, and maintain the mechanical properties of the disc [2].

Low back pain is a widespread health issue, affecting about 80% of people at least once in their lives [3]. Degenerative disc disease and lumbar disc herniation (LDH) are the most common causes [4]. LDH is defined as “localised or focal displacement of disc material beyond the limits of the disc space” [5]. They often cause spinal cord or nerve roots compression, resulting in pain, numbness, or weakness in dermatomal or myotomal distribution. 63% of symptomatic LDH will resolve within four to ten months of non-operative management [6]. Surgical excision of the offending disc material is necessary for patients who do not respond to conservative management. The annual cost to Medicare in the U.S. due to discectomy surgeries was estimated at $300 million (USD) in 2010 [7]. Following surgery, the incidence of recurrent LDH is 7–18% [8–10].

An ex-vivo herniation model is a mechanical protocol that applies forces to human or animal cadaver intervertebral discs (IVD) to create a herniation. Lumbar disc herniation models play a key role in research for two main reasons. Firstly, they serve as a valuable tool for researchers to understand the underlying mechanisms and pathophysiology of disc herniation. Secondly, they are essential for evaluating the efficacy of various implants, such as annulus closure devices [11] or nucleus replacement devices [12].

Numerous ex-vivo herniation models have been developed; however, robust validation against their clinical counterparts remains limited. One reason for this is that there are few collated datasets on the physiology of herniated discs. This review aims to address this issue, by collating clinical herniation data. Additionally, the review will use this data to evaluate whether any of the herniation models studied have created physiologically accurate herniations. Understanding which herniation models are physiologically accurate is important to improve our knowledge of how herniations occur; and whether they are appropriate to be used for testing implants.

## Methodology

Two comprehensive literature searches of MEDLINE were carried out in September 2023 (updated in February 2025 for new articles), in accordance with PRISMA guidelines. The first search aimed to find cadaveric ex-vivo herniation models, using the inclusion criteria of: utilising a mechanical model, single-level animal/human vertebra-disc-vertebra segments, and causing herniation to the disc. Studies were excluded if they were: clinical studies; not peer-reviewed; finite-element/ computational models; focused on small-animal models; focused only on specific regions of the disc; focused on diseases other than herniation; reusing a previous loading methodology; or unable to provide sufficient morphological details of the observed herniations.

**Fig. 1:**
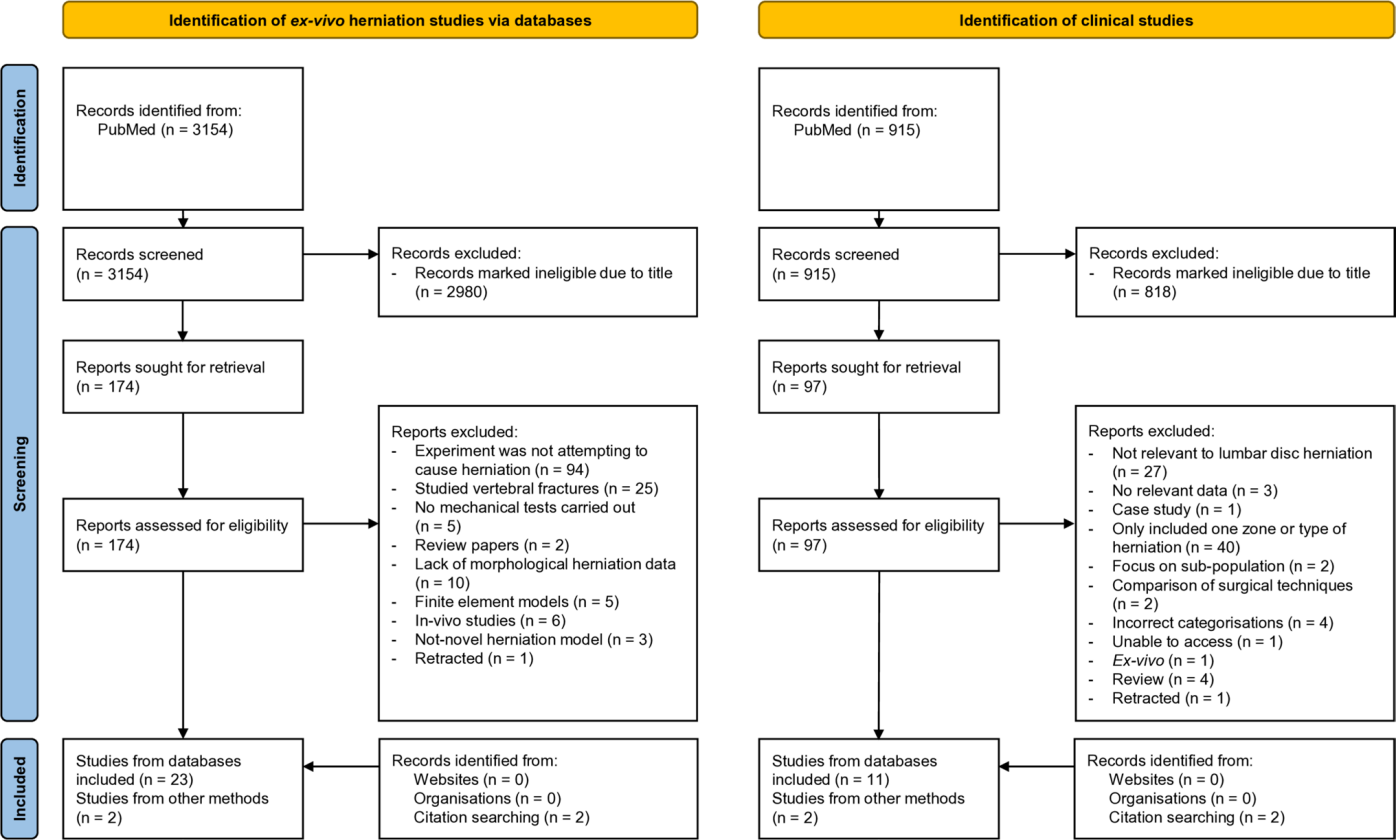
PRISMA flowchart for the literature search and screening strategy

The second search targeted clinical manuscripts detailing in-vivo herniation morphology. To be included, manuscripts needed to contain numerical data describing herniation morphology in patients. The exclusion criteria were: studies which focused only on one subgroup of herniation; were non-peer reviewed; were focused on endplate changes; classified herniations in a non-standard way; or did not look at herniation. The full search strategies are detailed in the supplementary information. One researcher carried out the search, using the PubMed search tool. Figure 1 illustrates the full study selection process.

**Fig. 2:**
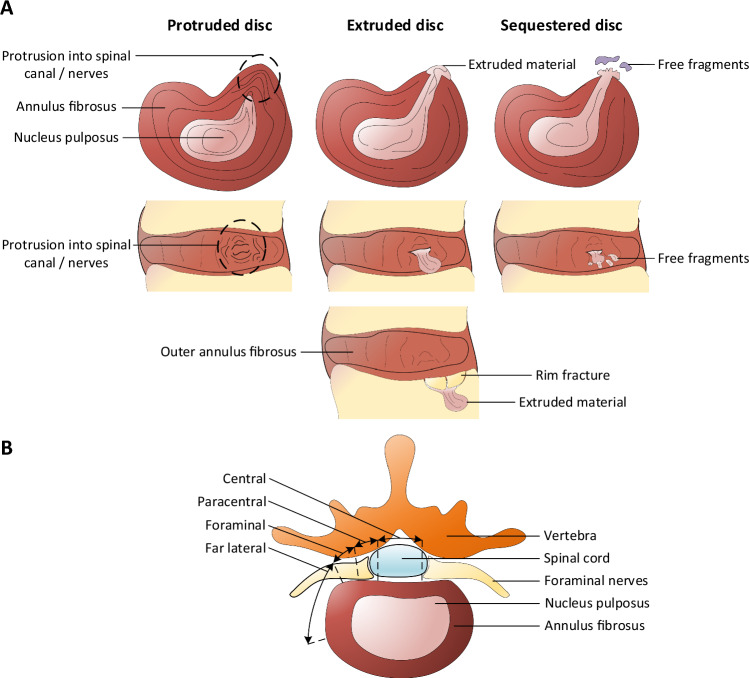
**A**: Types of herniation (protruded, extruded, and sequestered), shown in the transverse (top) plane and sagittal plane (bottom) of the disc. Two distinct morphologies of extruded discs are depicted: mid-annular (above) and endplate-junction failure (below). **B:** Zones of herniation within the disc (central, paracentral, foraminal and far lateral), shown in the transverse plane. The diagrams adhere to the standardised definitions set out by Fardon et al*.* (2014) [5]

The manuscripts identified from the ex-vivo herniation model search were then examined for: the type of loading; species and disc level; and the characterisations of the herniations produced. Where possible, herniations were classified by type (protrusion, extrusion, sequestration (Fig. 2A)), location (central, paracentral, foraminal, far lateral (Fig. 2B)) and any other notable morphologies of the herniations were also included. Numerical data for the herniation rate was taken directly from the studies, and where required simple calculations were carried out. From the second search, information about the morphology of herniated discs (*in-viv*o) was extracted from the studies. This included: the type; location; disc-level; and failure mode (EPJF or mid-annular tearing).

### Terminology

Due to a historic lack of any clear and unified definitions, many papers refer differently to herniated discs [13]. The biggest deviation from the nomenclature is the use of the term *‘prolapsed*’ as opposed to ‘*extruded*’ (or rarely, ‘*protruded*’). Furthermore, the term ‘bulge’ was frequently used to describe a herniation. If a distinction was made between a ‘bulge’ and a ‘protrusion’, then the bulge was not classified as a herniation; otherwise, it was included as a protrusion. If the herniation zone was classified as ‘posterolateral’ it was recorded as paracentral. However, if both ‘paracentral’ and ‘posterolateral’ were specified, it was categorised as foraminal (Fig. 2B) [13, 14].

## Results

The studies included are presented in Tables 1–7. 25 herniation models and 13 clinical studies met the inclusion criteria. Herniation models were categorised into three distinct loading types: complex ultimate compression (Table 1); cyclic (Table 2); and intradiscal pressurisation (Table 3). Mechanical tests involving pure axial compression resulted in vertebral fractures rather than herniation, which were excluded [15–17].

**Table 1:** Studies which caused IVD failure through complex ultimate compression. All studies compressed segments until failure. In studies with multiple groups, results were combined unless otherwise stated

Author (year) and *Last author*	Number of IVDs, *n*	Species	Rate	Flexion angle	Other complex loading mechanism/loading applied	Herniations/ failures seen? (% of total *n*)	Methods for determining herniation damage
Adams and Hutton (1982) [19]	61	Human	3 kN s^−1^	6—18°	Compressed in flexion, until approx. 8 kN. Upon non-failure, flexion angle was increased	43% of discs failed by extrusion (30%) or protrusion (13%). Mildly degenerated discs herniated most commonly (71%). Ages 30–59 most vulnerable to herniation (57%). Lower limits of flexion did not produce herniations	Bisected sample was visually inspected
Wade et al. (2014) [18] *ND Broom*	47	Ovine	2 and 40 mm min^−1^	10°	n/a	Herniations seen only in flexed, high-rate loading (54%). Both factors may be important for herniation to occur. 69% of herniations in central zone, 31% in foraminal	Microscopic analysis of sagittal sections for damage. Visually inspected during testing
Wade et al. (2015) [20] *ND Broom*	24	Ovine	40 and 400 mm min^−1^	10°	n/a	High-rate loading led to more herniations (83% vs 53%) and the endplate junction was also more likely to be injured (58% vs 16%). Fewer endplate fractures were seen at high-rate loading (17% vs 42%)	Microscopic analysis of sagittal, coronal and offset sections for macro-damage. Visually inspected during testing
Wade et al*.* (2017) [21] *ND Broom*	30	Ovine	40 mm min^−1^	7°	Lateral shear (20° lateral tilt)	33% of segments had vertebral fractures. Non-continuous tears were seen through the annulus (100%)	Microscopic analysis of sagittal, coronal and offset sections for macro-damage. Visually inspected during testing
Shan et al. (2017) [22] *ND Broom*	37	Ovine	400 mm min^−1^	7°	Lateral shear (20° lateral tilt)	Failure occurred much earlier than low-rate loading (17% lower). 53% had vertebral endplate fractures, and 30% had disc-endplate junction tearing. Was successful at creating direct radial annular tears (97%) which may result in extrusions	Microscopic analysis of sagittal, coronal and offset sections for macro-damage. Visually inspected during testing
van Heeswijk et al. (2018) [38] *ND Broom*	16	Ovine	40 mm min^−1^	10°	n/a	69% of discs extruded. Discs with larger annular puncture extruded through the whole (88%), whilst small puncture saw no extrusions through the wound itself. 75% of discs were found to have disrupted inner annulus	Transverse sections were visually inspected

**Table 2:** Studies which caused IVD failure through cyclic loading. In studies with multiple groups, results were combined unless otherwise stated

Author (year) *Last author*	Number of IVDs, *n*	Species	Maximum cycles	Freq./Hz	Compression *varied between samples	Further detail about loading	Herniations/ failures seen? (% of total* n*)	Methods for determining herniation damage
Brown et al*.* (1957) [15] *AJ Yorra*	1	Human	1000	18	Peak load 66 N	Unmeasured bending	Horizontal AF tears	Sagittal sections were visually inspected
Adams and Hutton (1983) [27]	41	Human	9600	0.66	Peak load 1.5–6 kN*	Flexion, 1° less than physiological max. Force applied based on weight, age, gender. Compressed to failure at end of cycles	7% annular protrusions, 93% vertebral body fractures occurred	Bisected through transverse plane and visually inspected
Adams and Hutton (1985) (Group B, D) [28]	30	Human	Until Failure	0.66	Sinusoid from 1.5 to 3kN*	Discs were flexed to a physiological limit, and cyclically tested. Every hour, the flexion angle was increased. In group D, a final compressive load was applied to force an extrusion to occur	21% of discs were extruded. Usually, the vertebral body broke. The group D disc extruded (*n* = 1)	Bisected through transverse plane and visually inspected
Wilder et al*.* (1988) [23] *JW Frymoyer*	Not stated	Human	6000	0.5	0.08–0.82 kN*	Constant axial load. Lateral bending and flexion torque applied cyclically, with unstated magnitudes, less than 34 Nm	75% of calf discs herniated. 1 Human spine failed via EPJF; it was stated to not herniate	Dissection post-testing and visually inspected
Gordon et al*.* (1991) [24] *EL Radin*	14	Human	38,000	1.5	Constant load of 1.3 kN	0–7° flexion, < 3° rotation	71% discs protruded, 29% extrusions	Pre- and post-test MRIs. Bisected through transverse plane and visually inspected
Callaghan et al*.* (2001) [25] *SM McGill*	26	Porcine	86,400	1	Constant load between 0.24 and 1.4 kN*	Subject flexed repetitively (max 29° to -12°, or 24 to -12 Nm) using angular or torque control	58% extrusions occurred, within the central, paracentral or foraminal zones	X-ray positive gel injected and imaged throughout testing
Drake et al*.* (2005) [26] *JP Callaghan*	18	Porcine	6000	1	Constant load between 0.24 and 1.4 kN*	Subject flexed repetitively (max 29° to -12°, or 24 to -12 Nm) using angular or torque control	73% extrusions occurred through AF. 50% had facet fracture, more often under axial torque	X-ray positive gel injected and imaged throughout testing
Tampier et al*.* (2007) [39] *SM McGill*	16	Porcine	4,400–14,400	1	Constant load of 1.5 kN	Subject flexed repetitively (15° to -2°)	50% herniated (no extrusions). 25% nucleus moved towards posterior AF, 25% no damage visible	X-ray positive gel injected and imaged. Dissected and viewed microscopically
Wilke et al*.* (2013) [11] *S Rath*	12	Human	100,000	5	Sinusoid from 0.1 to 0.6 kN	3–18 Nm torque cyclically applied. Torque was applied in both flexion and bending, whilst the disc was rotated 360° min^−1^	58% herniated. All extrusions. All mildly degenerated discs herniated	Sample exterior was visually inspected
Wilke et al*.* (2016) [29] *N Berger-Roscher*	8	Ovine	1200	0.5	0.8 kN constant	0 to 12° flexion, 0 to 9° lateral bending, 0 to 4° axial rotation	50% extruded, 25% protrusions. 50% endplate junction failures and 25% annular failures	Post-test UHF-MRI (11.7 T) for internal damage. Exterior visually inspected throughout testing
Berger-Roscher et al*.* (2017) [30] *H-J Wilke*	30	Ovine	1000	2	Sinusoid from 0 to 0.8 kN	0 to 13° flexion, 0 to 10° lateral bending, 0 to 4° axial rotation	57% endplate junction failures. 33% annular failures (no material became extruded)	Post-test UHF-MRI (11.7 T) for internal damage. Exterior visually inspected throughout testing
Zengerle et al*.* (2021) [31] *H-J Wilke*	12	Human	30	0.1	Maximum of 2 MPa, measured directly. Load held constant	Physiological movements: flexion (< 13°), rotation (< 1°) and lateral bending (< 6°) applied simultaneously	79% herniated. All extrusions. All mildly degenerated discs herniated	Post-test UHF-MRI (11.7 T) for internal damage. Exterior visually inspected throughout testing
Wade et al*.* (2022) [32] *H-J Wilke*	30	Ovine	1000	2	Sinusoid from 0 to 1.5 kN	0 to 13° flexion, 0 to 10° lateral bending, 0 to 4° axial rotation	13% discs extruded; AF was damaged in all (100%)	Post-test UHF-MRI (11.7 T) for internal damage. Exterior visually inspected throughout testing. Radial sections were analysed microscopically

**Table 3:** Studies which caused IVD failure through intradiscal pressurisation. In studies with multiple groups, results were combined unless otherwise stated

Author (year) *Last author*	Number of IVDs, *n*	Species	Posture	Mean failure pressure (MPa) *Failures via AF only	Herniations/ failures seen? Results exclude samples which failed primarily through the injection site	Methods for determining herniation damage
Schechtman et al*.* (2006) [33] *ND Broom*	25	Bovine (tail)	Neutral	18	Ruptures (100%) were seen and heard through the AF. Failure occurred entirely in the AF for 42% of samples, and 58% had failed through the EP	Blue dye was injected, and bisected sagittally for visual inspection
Veres et al*.* (2008) [33] *ND Broom*	9	Ovine	Neutral	14.1 13.2*	22% inferior EPs failures; 78% disc failures (43% extraforaminal, 57% central zone)	CT positive gel injected and visualised in 3D with micro-CT. Radial sections were analysed microscopically
Veres et al*.* (2009) [40] *ND Broom*	34	Ovine	7° or 10° flexion	10.3 9.9*	47% vertebrae failures; 53% disc failures (‘mostly’ central and paracentral)	CT positive gel injected and visualised in 3D with micro-CT. Radial sections were analysed microscopically
Veres et al*.* (2010) [36] *ND Broom*	23	Ovine	Neutral or 7° flexion	13.1	39% vertebra failures; 61% disc failures	CT positive gel injected and visualised in 3D with micro-CT. Radial sections were analysed microscopically
Veres et al*.* (2010) [35] *ND Broom*	25	Ovine	7° flexion and 2° axial rotation	7.9*	68% vertebrae failures; 32% disc failures (38% paracentral, 62% central zone)	CT positive gel injected and visualised in 3D with micro-CT. Radial sections were analysed microscopically

### Complex ultimate compression models (Table 1)

When subjected to compression, static flexion, and shear forces, over 40% of segments herniated [18, 19]; with higher loading rates (40 vs 400 mm min^−1^) leading to an increased herniation rate (58% vs 83%), and more damage to the endplate structures (16% vs 58%) [20]. 95% + of segments compressed in this way also developed radial tears of AF [21, 22]. Overall, flexion (6° +) was the key factor for herniation, alongside a high rate of loading (maximum loading reached within ~ 10 s).

### Cyclic models (Table 2)

Studies applied different types of cyclic load. Some applied cyclic flexions (with static compression) [23–26]; or cyclic compressions (with static flexion) [15, 27, 28]; whilst others have utilised a combination of cyclic rotations and compression [11, 29–32]. Cycle counts ranged from 30 – 100,000, and test periods from 1 min to 24 h. All cyclic models were able to cause herniations, however, cyclic compressions were the least effective resulting in a maximum of just 21% of discs herniating [28]. Flexion exceeding 7° was a common factor in all models, resulting in over 50% of samples herniating [11, 23–26, 29, 31].

### Intradiscal pressurisation herniation models (Table 3)

Hydrated silica-gel was injected into IVDs at pressures of up to 55 MPa [33]. Flexing samples (7° +), decreases the pressure required for failure (14 vs 8 MPa) [34, 35]; whilst applying torsion (2°) leads to fewer herniations (64 vs. 32%) and less AF rupturing (18 vs. 8%) [35]. Higher rates of loading increase the risk of endplate damage [36].

### Loading type comparisons

**Fig. 3:**
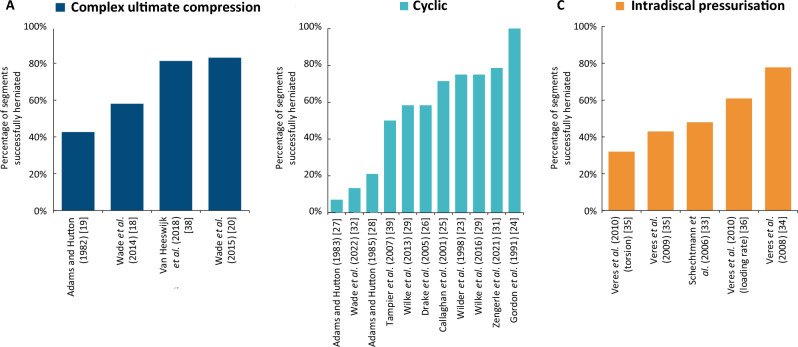
Percentage of herniations created in each study. Studies which obtained no herniations were excluded. **A**: Complex ultimate compression. The result for Adams and Hutton (1982) only includes the mildly degenerate discs. **B**: Cyclic models. The result for: Wilke et al*.* (2013) only includes the mildly degenerate discs tested; and Adams and Hutton (1985) only includes results reported from *method B* within the study (due to the low herniation rate in *method A* and *C*). **C**: Intradiscal pressurisation models. Veres et al*.* (2008, 2009, 2010 [torsion], 2010 [loading rate]) discs were classed as herniated when they found ruptured AF, as this implied a pathway for extrusion

Figure 3 illustrates that all three of these loading types can produce herniations, at rates of up to 80%. Whilst overall herniation rates have been found to be similar, cyclic models often result in more progressive damage, such as annulus delamination and gradual extrusions [25, 27], whilst ultimate complex compression models result in a higher likelihood of fractures and ‘sudden’ herniations [18, 19]. Both herniation morphologies are physiologically relevant and closely resemble mid-annular or endplate junction failure (EPJF)-type herniations [37]. Intradiscal pressurisation models produce failures that illustrate how NP material tracks through the disc and highlight structural failures throughout the disc, prior to extrusion [34]. Only one model incorporated multiple loading types applied in a sequential manner, potentially leading to a higher rate of herniations [28].

### Clinical data

Extrusions account for the highest proportion of herniations at 50% (Table 4). Protrusions are the second most prevalent at 34%, however, three out of five studies report more protrusions than extrusions [37, 41–43], suggesting that the rate of extrusion and protrusions are likely comparable. Sequestration is the least common form of herniation, observed in 14% of patients.

**Table 4:** Summary of the types of herniation found clinically

Author	*n*	Protrusion/%	Extrusion/%	Sequestered/%
Brock et al*.* (1992) [45]	350	28.5	43	28.5
Ohshima et al*.* (1993) [46]	471	29	65	6
Rajasekaran et al*.* (2013) [37]	181	45	33	10
Sahoo et al*.* (2017) [43]	66	57	20	23
Yaman et al*.* (2017) [47]	126	53	40	6
Overall	1172	36	50	14

Paracentral herniations are the most frequently documented, making up 61% of cases (Table 5), followed by central (25%) and foraminal (12%) herniations.

**Table 5:** Zones of herniation seen clinically

Author	*n*	Central/%	Paracentral/sub-articular/%	Foraminal/%	Extraforaminal/far lateral/%
Ebeling and Reulen (1992) [48]	131	5	63	20	11
Knop-Jergas et al*.* (1996) [49]	60	32	42	25	1.2
Rajasekaran et al*.* (2013) [37]	181	9	83	6	2
Mérot et al*.* (2014) [50]	59	53	5	34	8
Dutta et al*.* (2016) [51]	51	27	65	8	0
Krishnan et al*.* (2017) [52]	140	39	31	4	27
Overall	622	23	54	13	10

Certain disc levels, particularly L4-L5 (49%) and L5-S1 (42%) (Table 6) are more prone to herniation due to increased physiological forces and differing neutral positions.

**Table 6:** Clinical data of herniated disc level

Author	*n*	Disc Level/%				
		L1-L2	L2-L3	L3-L4	L4-L5	L5-S1
Ebeling and Reulen (1992) [48]	131	0	3	5	48	44
Knop-Jergas et al*.* (1996) [49]	80	0	0	0	51	49
Wittenberg et al*.* (1998) [53]	54	0	0	1.9	35	63
Choi et al*.* (2007) [54]	68	0	0	3	53	44
Rajasekaran et al*.* (2013) [37]	181	0	1.7	6	49	43
Mérot et al*.* (2014) [50]	59	0	0	17	41	42
Dutta et al*.* (2016) [51]	56	0	0	13	48	39
Krishnan et al*.* (2017) [52]	140	1.4	4	14	56	25
Yaman et al*.* (2017) [47]	600	0.7	3	7	48	42
Sahoo et al*.* (2017) [43]	66	0	0	9	53	38
Overall	1435	0	2	7	49	42

Clinicians have reported inconsistent findings regarding the structural failure point, with AF failure ranging from 35 to 81% and endplate junction failure (EPJF) between 19 and 68% (Table 7). The higher EPJF rate has been hypothesised to result from a potentially lower bone mineral density in the studied population [44].

**Table 7:** Failure point of disc during herniation seen clinically. No overall row was included due to the limited number of studies and the high variability within the data

Author	*n*	Other Failure (*e.g.*Annular)/%	Endplate Junction Failure/%
Rajasekaran et al*.* (2013) [37]	181	35	65
Sahoo et al*.* (2017) [43]	66	32	68
O’Neill et al*.* (2020) [44]	26	81	19

### Comparisons between models and the clinical data

The morphological data found within the ex-vivo herniation studies (type, zone, level) were compared to clinical data. Nine studies provided sufficient information on herniation type, and nine offered enough detail to interpret the herniation zones. However, only three studies were suitable for analysing disc level, primarily due to the exclusion of animal studies.

#### Zone of herniation (Fig. 4A)

**Fig. 4:**
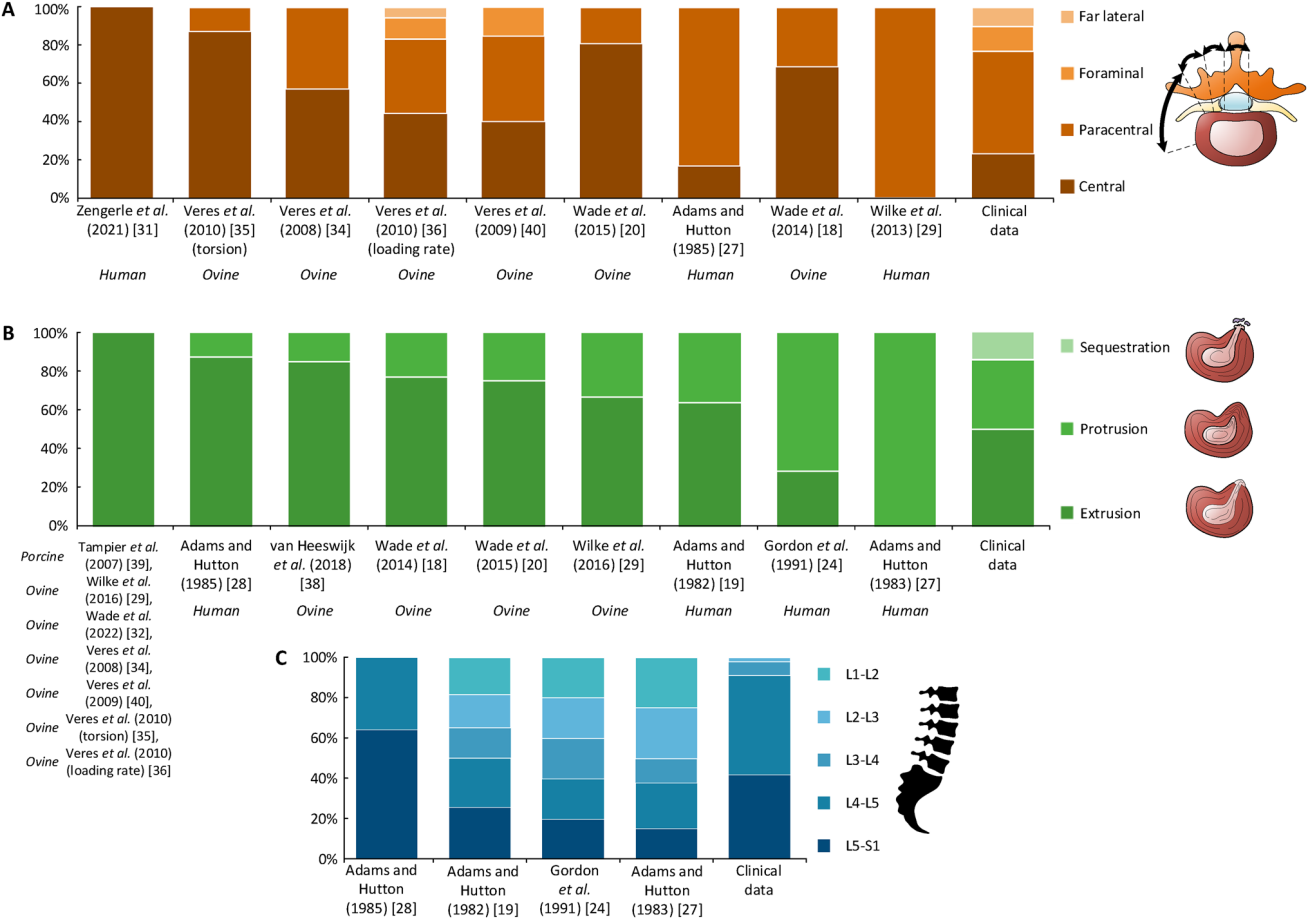
**A:** Percentage of type of herniation created in each study. Clinical data from Table 4. Species in ex-vivo studies given in italics. **B:** Comparison of the herniation zone seen clinically and in ex-vivo herniation models. Clinical data from Table 5. Species in ex-vivo studies given in italics. **C:** Comparison of herniation disc level in clinical and ex-vivo human cadaveric data. The ex-vivo data was made by calculating a percentage herniated for each disc level tested, and then these percentages were normalised to 100%, to create a directly comparable result to the patient data. Data from Adams and Hutton (1983) was taken from the a*nnular distorted* discs. Clinical data from Table 6

Depending on the model used, the majority of herniations occurred in the central or paracentral zone. This is consistent with data from patients, which shows that 54% of herniations occur in the paracentral zone and over 75% in either the central or paracentral zones. However, herniation zones were less variable within each model compared to clinical observations. This may be due to procedures like annulotomy, which can cause herniations to develop in a predefined zone.

#### Type of herniation (Fig. 4B)

The majority (87%) of ex-vivo models demonstrate extrusion as the most common type of herniation*.* Sequestration was not seen to occur due to the absence of surrounding tissue preventing sequestration from occurring. Additionally, models often produced herniations of the same type in over 75% of cases, likely due to two factors: 1) consistent loading, which leads to more controlled herniations compared to clinical cases, and 2) the use of annulotomies, which create a clear pathway for extrusion.

#### Disc level (Fig. 4C)

Ex-vivo models can induce herniation at any disc level, whereas clinically, the majority (85% +) of herniations occur at L4-L5 and L5-S1. This may be because hyper-physiological forces and flexions are applied to ex-vivo upper lumbar spine segments. Several studies excluded L5-S1 discs [11, 23], which may have led to an oversight of important herniation mechanisms at this level.

#### Clinical data comparison summary

No single herniation model was found to replicate the distribution of herniation zones, types, or affected disc levels observed in patients. However, some models demonstrate similarity in certain aspects, for example, the model from Wilke et al*.* (2016) [29] found that 66% of segments underwent EPJF, similar to the clinical findings of Rajasekaran et al*.* (2013) (65%) [37].

When discrepancies were observed, these may be accounted for by several factors: (1) Ex-vivo models can control loading conditions, ensuring consistent loads are applied, which contrasts with the uncontrolled, complex, and dynamic biological environment seen (2) Ex-vivo models lack biological processes, such as discs becoming repeatedly damaged and healing over time, which may result in different distributions of morphologies. (3) While ex-vivo experiments can select for segments based on various biological factors—such as age, degeneration, weight, and smoking— clinically, it is more challenging to account for all of these variables.

While these differences suggest greater variability in herniation morphologies in clinical settings, this does not mean that the more consistent patterns observed in herniation models are not representative of clinical cases.

It should be noted that the demographics of the clinical and ex-vivo data sets were not matched, and differences in factors such as age, sex, and disc level may also have contributed to discrepancies in herniation data observed between the two.

## Discussion

The results provide a snapshot of how herniation models replicate clinical morphologies (Fig. 4A–C); and often individual models produce only a single type or zone of herniation. The primary aim of many studies is to understand the underlying mechanisms of herniation progression. Achieving this requires making inferences from observed damage. This indicates that each model may be limited to replicating a specific morphology, and mechanistic inferences should be made cautiously and within the constraints of each model’s design.

### Methods for herniation inference

Understanding the mechanisms of disc herniation remains a significant challenge in spinal research. Studies have employed various methods to demonstrate damage within discs (Table 1, 2 and 3). Most manuscripts rely on various imaging (high-resolution MRI; microstructural analyses) methods utilised after mechanical loading to assess the resulting damage. The damaged observed is seen to vary in severity and presentation across discs. Inferences can then be made as to how the herniation progresses through the observed stages of damage allowing mechanistic hypotheses for how in-vivo herniation occurs to be formulated [18–20, 22, 28, 30, 32]. However, this approach has limited use, as it is unable to demonstrate how damage progresses; nor does it reveal if a certain type of damage is required for the next stage of the process to occur.

Herniation is considered to be a progressive disease [55], although it may occur suddenly, likely due to pre-existing damage to the disc [28]. Given the disc’s limited healing capacity [56], it may be possible to study herniation progression using a mechanical model, and non-invasive techniques may be able to be used to monitor how damage occurs. For instance, ultra-high field MRI may be used in ex-vivo testing to give a snapshot of the damage prior to, or mid-way through testing [32]. This technique may be used to demonstrate how failure cascades trough the disc into a complete disc herniation. However, lacks the spatial resolution to pick up failures seen under microstructural analysis. Additionally, data collected during loading, such as force–displacement curves or X-ray images, should be cross-referenced with the observed damage. This will enhance our understanding of failure mechanisms, enabling correlations to be made between specific loading conditions with the damage that occurred. Whilst not considered within the scope of this review; the use of finite-element modelling can also provide useful insights as to areas of high stress within the discs; and give indications of where failure may be likely to occur [57].

### Mechanistic comparison

Despite the variety of models that have been employed, no single model has successfully replicated the wide distribution of morphologies seen during clinical herniation. This discrepancy may be attributed to the differences in timescale and the progressive nature of herniation in clinical settings compared to controlled laboratory environments.

The replication of a herniation extends beyond observing the morphology of the injury itself, but also to the pathway and partial failures which occur prior to herniation. By studying the regions of the disc that fail first under specific loading conditions of the disc, and the progression of damage in lab settings, we may gain valuable insights into the mechanisms of herniation. This knowledge may inform better management strategies for patients at risk of herniated discs. Additionally, care must be taken when generalising failures within the disc; as these are likely to be affected not only by the loading applied but also specimen factors like age, disc degeneration, smoking [58].

Our clinical understanding of herniation mechanisms is limited due to the inherent challenges in studying this condition. However, insights can be gained through histopathological and clinical observation studies. Histological analyses of excised disc material have revealed the presence of AF tissue interspersed with NP material, as well as EP tissue [59, 60]. This suggests that the damage during herniation may involve the migration of NP material through the disc; with tears occurring within the AF; and EP being torn from the vertebrae itself (EPJF). These results agree with clinical observation studies which found herniation occurred through both EPJF and mid-annular tearing [37, 44, 59]. It is rare for herniation to occur as a comorbidity alongside vertebral fractures; although cases can occur in rare circumstances [61, 62]. Therefore, a mechanistically accurate herniation model may find NP movement or extrusion, EPJFs (including rim fractures), and mid-annular tearing, without severe vertebral fractures [30].

Ex-vivo models have led to several proposed mechanisms of herniation. For instance, Adams and Hutton (1983) were the first to propose a mechanistic hypothesis for how damage that leads to herniation occurs under fatigue loading [28]. They propose that the process begins with compaction of the posterolateral AF before the NP begins to break through lamellae, eventually rupturing the outer layers of the disc. Conversely to mid-annular failure, Berger-Roscher et al*.* (2017) inferred from their complex-loading study that the initial failure point may be a fracture within the vertebra, with the bony endplate and the cartilaginous endplate remaining intact away from the point of failure [30]. They suggest that the failed vertebra opens a pathway for nucleus tissue to extrude through. It is also possible that the failed vertebra may lead to an AF protrusion, as the tension in the annulus fibrosus may be partially released.

Complex posture ultimate compression models (Wade et al*.* (2017), Shan et al*.* (2017)) showed that the initial failure may occur in the mid-AF region: at the endplate junction under low loading rates, or through a mid-annular tear at high loading rates [21, 22]. It was hypothesised that the damage could then propagate toward the outer-AF, leading to an extrusion-type failure. The mechanisms proposed by the above models all seem plausible; however, due to the loading applied in each of these models being non-physiological, it is difficult to infer whether these proposed mechanisms could occur in-vivo. Due to differences in the types of herniations seen when different loading modes are applied, it may be concluded that the progression of a herniation in-vivo may be affected by the activities undertaken by an individual, such as playing professional football [63].

Whilst not proposing an overall mechanism, individual forms of disc damage which may lead to herniation have been studied. One form of this is annular tearing, which can occur across adjacent or alternating AF layers [22, 34, 40]. Tearing of alternating fibres is expected to occur when the disc is flexed and rotated, creating a stress differential between the different fibre directions of each layer of AF [22, 35]. Additionally, tracking of NP tissue through layers of AF has been seen (both circumferentially, and radially) [34]. The circumferential travel of NP tissue may suggest that the pressurised NP only extrudes upon finding a weak spot in the AF [39].

There are several limitations to the mechanistic hypotheses of herniation. Firstly, the progression of the herniation had to be inferred from discs with varying degrees of damage, assessed only after loading was applied. Whilst several studies have applied cyclic loads to discs (Table 3), none of these studies have been able to demonstrate the progression of structural damage of herniation in a single disc.

Secondly, herniation models typically apply a continuous load until failure occurs. However, this approach fails to consider the discs’ limited healing capacity, as evidenced by 80% of herniations improving without surgical intervention [64]. Since this healing process cannot be reflected in ex-vivo models, it is unclear whether the mechanisms derived from mechanical tests accurately represent those occurring in-vivo. However, due to the avascularity of the intervertebral disc and the long duration of healing periods, ex-vivo testing remains well accepted for identifying trends and understanding disc failure mechanisms [65]. Finally, due to the limitations of acquiring herniation data from patients, it is challenging to determine which mechanisms are most accurate.

While this review provides a qualitative comparison between ex-vivo and clinical data, it highlights the current limitations within the field. The absence of standardised metrics, coupled with variations in experimental design and sample characteristics, complicates direct statistical comparisons. Moving forward, future research should explore the development of a quantitative framework for comparing herniation mechanisms, though achieving this will require coordinated efforts across multiple research groups.

### Considerations for model choice in herniation mechanism research

Herniation models should accurately replicate physiological damage. Since some discs are unlikely to herniate in-vivo (Table 6), and 20% of people never experience back pain or herniation [4], applying supra-physiological loads may therefore be necessary to induce a realistic herniation response within a suitable timeframe.

Researchers must also consider the physiological relevance of the loading applied in herniation models to accurately simulate a physiological herniation mechanism. For example, using flexion combined with lateral bending creates asymmetric loads that may affect the posterior and posterolateral area of the disc, similar to the impact of picking up a weight with poor spinal posture [66].

These loading conditions also increase disc pressure [67], a factor that has been shown to contribute to herniation [68]; and this premise is further supported by the observation of increased lumbar disc herniations in astronauts returning from space, where low-gravity driven hyper-swollen discs are more prone to herniation during normal activities [69].

Animal models are often used as substitutes for human discs, but differences in geometry, degeneration, and the NP-to-AF ratio make it unclear how their herniation mechanisms relate to humans [70]. These factors affect stress distribution, altering the areas most prone to damage [71, 72]. Therefore, caution must be applied when making inferences of herniation mechanisms from animal models. While animal models are useful and each species likely provides unique insights, human models—particularly non-degenerate discs—are generally considered the most clinically relevant when available. Given the variability across species, a comparative study of animal models could help determine the limitations of these discs in herniation, and provide evidence for which discs best replicate human herniation mechanisms.

It is currently challenging to identify a single model as the most clinically relevant, due to numerous confounding variables (choice of loading, tissue degeneration grade, and species selection) between studies and the variety of outcome measures reported. A valuable direction for future research would be the development of a standardised testing approach to determine the conditions most appropriate for modelling different types of herniation.

### Clinical recommendations

Clinical guidelines recommend exercise for managing low back pain and preventing disc herniations [73]. Our findings on herniation modelling supports this; however, since herniations were successfully induced using both complex compressions and cyclic loads, we suggest that patients experiencing lower back pain flare-ups avoid lifting (using proper posture if necessary) and refrain from repetitive activities like running or extensive walking.

## Conclusions

This review examines clinical herniation data to evaluate the physiological accuracy of ex-vivo lumbar disc herniation models. Our findings suggest that typical clinical herniations involve extruded discs with rim-fracture EPJF at the L4-L5 level in the paracentral zone, though there is considerable variation in these parameters. In contrast, herniation models generally produce more specific and repeatable outcomes. Models from all loading types (complex posture ultimate compression, cyclic loading, intradiscal pressurisation) successfully induced herniations, each providing valuable mechanistic insights into disc failure. Validating the herniation models against clinical cases is challenging due to the limitations of in-vivo studies. Therefore, when selecting a herniation model, researchers must consider the physiological relevance of the applied load, as well as the differences between animal and human discs, which may influence the resulting damage and inferred mechanisms. Future research should focus on understanding herniation progression to identify initiating factors, which may help improve preventative strategies.

## Supplementary Information

Below is the link to the electronic supplementary material.

Supplementary file1 (DOCX 15 KB)

## Data Availability

All search data generated or analysed during this study are included in the published article, and the search criteria can be found in the supplementary information files.
