# Supplementary material for: Lumbar disc herniation modelling: a review of ex-vivo mechanical models and a comparison with clinical data
Source: Eur Spine J. 2025 Jun 25;34(10):4353–68. doi: 10.1007/s00586-025-09054-x (PMC12496270; doi:10.1007/s00586-025-09054-x)
Supplement: Supplementary file 1 — Supplementary file1 (DOCX 15 KB) [file 586_2025_9054_MOESM1_ESM.docx]

**Appendix A: Search Strategy**

The search for the herniation model was written to ensure that the manuscripts were: spinal disc related; applied to both animal and human models; incorporated all loading mechanisms; and that the segments were tested until a failure had occurred. Then, the title-exclusion words were used to remove studies using: finite-element models; small-animal models; studying diseases other than lumbar herniation and vertebral or endplate failure; reviews; clinical studies and medical device studies; and therapeutic/tissue engineering studies. Search one was as follows:

(spine AND (lumbar OR vertebra OR "intervertebral disc" OR "intervertebral joint"))

AND (human OR cadaver OR animal OR ovine OR bovine OR porcine)

AND (loading OR mechanical OR biomechanical)

AND (compression OR complex OR cyclic OR repetitive OR flexion OR extension OR bending OR pressurisation OR torsion OR torque

OR rotation OR shear)

AND (failure OR injury OR herniation OR "disc herniation" OR damage OR fracture)

NOT ("finite element"[ti] OR FEM[ti] OR "in-silico"[ti] OR numerical[ti] OR simulated[ti] OR computation[ti] OR "small animal"[ti] OR rats[ti] OR rat[ti] OR mice[ti] OR rabbit[ti] OR spondylolysis[ti] OR kyphoplasty[ti] OR vertebroplasty[ti] OR arthroplasty[ti] OR osteoporosis[ti] OR osteopenia[ti] OR cement[ti] OR prophylactic[ti] OR screw[ti] OR bone[ti] OR cortical[ti] OR trabecular[ti] OR fixation[ti] OR management[ti] OR cervical[ti] OR review[ti] OR "clinical trial"[ti] OR clinicians[ti] OR patients[ti] OR traction[ti] OR brace[ti] OR "total disc replacement"[ti] OR "interbody fusion cages"[ti] OR fusion[ti] OR fusions[ti] OR "in-vivo"[ti] OR instrumentation[ti]

OR inflammation[ti] OR "TNF-alpha"[ti] OR "hormone"[ti] OR hydrogel[ti] OR “cell-seeded”[ti] OR inhibitor[ti] OR matrix[ti])

Search two was written to find studies which reported on the morphology of herniation. The clinical-data search aimed to gather data to make conclusions about the morphology of herniation. The search looked for spinal disc herniation clinical studies, which had data in them about how herniation occurs. Title-words were used to exclude manuscripts which were: case studies; reviews; animal models; or unrelated to the morphology of herniation. Search two was as follows:

herniation

AND spine

AND clinical

AND (prospective OR predictors OR anatomy OR morphology OR typical OR location)

AND (EPJF OR zone OR central OR paracentral OR lateral OR foraminal OR median OR paramedian OR endplate)

NOT (immune[ti] OR pain[ti] OR "surgical approach"[ti] OR discography[ti] OR review[ti] OR "case study"[ti] OR animals[ti] OR dogs[ti] OR pigs[ti] OR sheep[ti])
